# Association between cancer history and second-generation drug-eluting stent thrombosis: insights from the REAL-ST registry

**DOI:** 10.1186/s12959-023-00503-5

**Published:** 2023-05-24

**Authors:** Tomoyo Hamana, Hiromasa Otake, Shoichi Kuramitsu, Tomohiro Shinozaki, Masanobu Ohya, Kazunori Horie, Hiroyoshi Kawamoto, Futoshi Yamanaka, Masahiro Natsuaki, Hiroki Shiomi, Gaku Nakazawa, Kenji Ando, Kazushige Kadota, Shigeru Saito, Takeshi Kimura, on behalf of the REAL-ST Registry Investigators

**Affiliations:** 1 Division of Cardiology, Department of Internal Medicine, Kobe University Graduates School of Medicine, Kobe, Japan; 2 Department of Cardiology, Kokura Memorial Hospital, 3-2-1 Asano, Kokurakita-Ku, Kitakyushu, 802-8555 Japan; 3 Department of Information and Computer Technology, Faculty of Engineering, Tokyo University of Science, Tokyo, Japan; 4 Department of Cardiology, Kurashiki Central Hospital, Kurashiki, Japan; 5 Department of Cardiovascular Medicine, Sendai Kousei Hospital, Sendai, Japan; 6 Department of Cardiology, New Tokyo Hospital, Chiba, Japan; 7 Division of Cardiology and Catheterization Laboratories, Shonan Kamakura General Hospital, Kanagawa, Japan; 8 Department of Cardiovascular Medicine, Saga University, Saga, Japan; 9 Department of Cardiovascular Medicine, Graduate School of Medicine, Kyoto University, Kyoto, Japan; 10 Department of Cardiology, Kindai University Faculty of Medicine, Osaka, Japan; 11 Department of Cardiology, Hirakata Kohsai Hospital, Osaka, Japan

**Keywords:** Stent thrombosis, Cancer-associated thrombosis, Drug-eluting stent, Percutaneous coronary intervention

## Abstract

**Background:**

Cancer-associated thrombosis is a frequent complication of cancer; however, little evidence is available regarding the association between cancer history and coronary artery stent thrombosis (ST). We aimed to investigate the relationship between cancer history and second-generation drug-eluting stent thrombosis (G2-ST).

**Methods:**

From the REAL-ST (Retrospective Multicenter Registry of ST After First- and Second-Generation Drug-Eluting Stent Implantation) registry, this study evaluated 1265 patients (G2- ST cases, *n* = 253; controls, *n* = 1012) with cancer-related information available.

**Results:**

The prevalence of patients with cancer history was higher (12.3% vs. 8.5%, *p* = 0.065), and that of currently diagnosed and currently treated cancer was significantly higher in ST cases than controls (3.6% vs. 1.4%, *p* = 0.021; 3.2% vs. 1.3%, *p* = 0.037, respectively). Multivariable logistic regression analysis revealed that cancer history was associated with late ST (odds ratio [OR]: 2.80, 95% confidence intervals [CI]: 0.92–8.55, *p* = 0.071) and very late ST (OR: 2.40, 95% CI: 1.02-5.65, *p* = 0.046), but not with early ST (OR: 1.01, 95% CI: 0.51-2.00, *p* = 0.97). During the median follow-up period of 872 days after the index ST events, patients with cancer history showed a higher mortality than those without, among both ST cases (hazard ratio [HR]: 1.93, 95% CI: 1.06-3.51, *p* = 0.031) and controls (HR: 1.93, 95% CI: 1.09-3.40, *p* = 0.023).

**Conclusion:**

A post hoc analysis of REAL-ST registry revealed that patients with G2-ST had a higher prevalence of currently diagnosed and currently treated cancer. Notably, cancer history was associated with the occurrence of late and very late ST, but not with early ST.

**Supplementary Information:**

The online version contains supplementary material available at 10.1186/s12959-023-00503-5.

## Introduction

Stent thrombosis (ST) is a serious complication after percutaneous coronary intervention (PCI), manifesting as myocardial infarction (MI) or even as cardiac death [1]. Second-generation drug-eluting stents (G2-DES) have reduced the incidence of ST compared to that with first-generation drug-eluting stents (G1-DES), while it remains an unsolved issue even in the current DES era.

Cancer-associated thrombosis is a frequent complication of cancer, contributing to the second-leading cause of mortality in cancer patients [2]. The underlying mechanism is an activation of the coagulation cascade and platelet activity via the production of microparticles and secreted factors and inflammatory cytokines by cancer cells or anticancer treatments themselves [3]. Traditionally, cancer-associated thrombosis has been focused on venous thromboembolism. However, recent studies have demonstrated that arterial thromboembolism, including MI and stroke, is a non-negligible complication in cancer patients [4]. Cancer and cardiovascular disease often coexist with the aging population and share common risk factors; patients concomitant with cancer and cardiovascular disease have worse survival rates than those with cancer alone [5, 6]. Patients with coronary artery disease have an increased risk of developing cancer [7], while little evidence is available regarding the relationship between cancer and ST after G2-DES implantation. The present study aimed to investigate the impact of cancer history on the incidence of ST and subsequent outcomes after ST using the REAL-ST (retrospective multicenter registry of patients with ST after G1-and G2-DES implantation) registry database [8].

## Methods

### Study design

This study was a post hoc analysis of the REAL-ST registry, a retrospective multicenter registry of patients with definite ST after G1- and G2-DES implantation at 46 Japanese PCI institutions. The study design and main results of the REAL-ST registry have been reported elsewhere [8]. Briefly, patients who fulfilled the following criteria were enrolled: (1) who underwent PCI with G1-DES from April 2004 to December 2013 or G2-DES from February 2010 to December 2015; and (2) who had definite ST of G1- or G2-DES from April 2004 to March 2017. For each definite G2-DES thrombosis (G2-ST) case in each participating center, we retrospectively identified two consecutive cases of PCI with G2-DES, immediately before and after the initial PCI procedure of the definite G2-ST case, who had not experienced any definite ST, as control patients.

For the present study, we analyzed patients (1) who experienced definite G2-ST and their corresponding control patient, and (2) whose cancer-related information was available. Patients with insufficient data on cancer, missing medical records due to their expired preservation period, and missing data on corresponding control patients were excluded from this study. The study protocol was approved by the ethics committees of all participating centers. The study was conducted in accordance with the Declaration of Helsinki and its amendments. Written informed consent was waived due to the retrospective study design. This study was registered with the UMIN Clinical Trials Registry (UMIN000025181).

### Cancer history definition and clinical data collection

Patients with cancer were defined as having a cancer history at the index ST events. According to the timing of the cancer diagnosis, cancer patients were categorized as those with currently diagnosed cancer (≤ 1 year before the index ST events) or those with previously diagnosed cancer (> 1 year before the index ST events). We also categorized cancer patients as those with currently treated cancer (≤ 1 year before the index ST events) or those with previously treated cancer (> 1 year before the index ST events), according to the timing of cancer treatment. In addition to baseline patient and lesion characteristics, we retrospectively recorded the cancer type and stage, and treatment details (i.e., surgical, radiation, chemotherapy, and hormone therapy).

### Clinical endpoints and definitions

The main objective of this study was to investigate the association of cancer history with the incidence of G2-ST and mortality after G2-ST occurrence. Cardiac death, major adverse cardiovascular events (MACE), non-cardiac death, target lesion revascularization (TLR), recurrent ST, and bleeding events were also assessed. Death was regarded as cardiac death unless other noncardiac death could be identified. MACE was defined as a composite of all-cause death, non-fatal MI, and TLR. TLR was defined as a repeated PCI or repeated coronary artery bypass graft at the target lesion. Recurrent ST was defined as the recurrence of acute coronary syndrome and angiographic evidence of thrombus in the same stent [9]. Bleeding was defined as a Bleeding Academic Research Consortium (BARC) score of 1 to 3 or BARC 5 bleeding [10]. Clinical events were ascertained from a review of medical records and confirmed by direct contact with the patients, their families, or physicians. Patients who were lost to follow-up were censored on the last day with follow-up information. Follow-up intervals were calculated from the day of the index ST events.

### Statistical analysis

This is a case-control study for definite ST versus non-definite ST among patients who received PCI with G2-DES. Categorical variables are presented as number (percentage) and continuous variables are expressed as mean ± standard deviation or median (lower and upper qualities) Cancer types, stages, and treatments were compared between G2-ST cases and controls using the chi-squared test. We conducted multivariable logistic regression analyses to investigate factors (including cancer information) independently associated with definite ST, early ST (EST; ST within 30 days), late ST (LST; ST from 31 to 365 days), and very late ST (VLST; ST > 1 year) [11]. We used two multivariable models with cancer-related information, in addition to the same clinically relevant factors used in a previous REAL-ST study, as covariates (listed in Table 1) [8]: model 1 included clinical factors and any cancer history, and model 2 included clinical factors (same as those in model 1) and cancer-related information classified by the timing of the cancer diagnosis/treatment (currently diagnosed/treated, previously diagnosed/treated, and non-cancer).

**Table 1:** Baseline clinical characteristics

	**ST** **(*n* = 253)**	**Controls** **(*n* = 1012)**	**EST** **(*n* = 142)**	**Controls** **(*n* = 568)**	**LST** **(*n* = 53)**	**Controls** **(*n* = 212)**	**VLST** **(*n* = 58)**	**Controls** **(*n* = 232)**
***Patient characteristics***								
Age, yrs^a^	67.9 ± 10.9	69.7 ± 10.7	68.0 ± 10.6	69.5 ± 10.5	66.4 ± 12.0	69.5 ± 11.5	68.9 ± 10.5	70.1 ± 10.2
Male sex^a^	196 (77.5)	768 (75.9)	115 (81.0)	437 (75.2)	40 (75.5)	165 (77.8)	41 (70.7)	176 (75.9)
BMI, kg/m^2a^	23.6 ± 3.6	23.8 ± 3.6	23.9 ± 3.5	23.8 ± 3.4	23.7 ± 4.2	24.0 ± 3.5	23.0 ± 3.2	23.7 ± 3.9
Hypertension^a^	201 (79.4)	834 (82.4)	112 (78.9)	469 (82.6)	41 (77.4)	176 (83.0)	48 (82.8)	189 (81.5)
Diabetes mellitus^a^	127 (50.2)	465 (45.9)	67 (47.2)	248 (43.7)	33 (62.3)	107 (50.5)	27 (46.6)	110 (47.4)
Dyslipidemia^a^	209 (82.6)	846 (83.6)	119 (83.8)	481 (84.7)	44 (83.0)	181 (85.4)	46 (79.3)	184 (79.3)
Current smoker^a^	68 (27.0)	205 (20.3)	45 (31.9)	129 (22.8)	9 (17.0)	29 (13.7)	14 (24.1)	47 (20.3)
Hemodialysis^a^	38 (15.0)	73 (7.2)	9 (6.3)	32 (5.6)	21 (39.6)	22 (10.4)	8 (13.8)	19 (8.2)
Prior myocardial infarction	92 (36.4)	289 (28.6)	50 (35.2)	160 (28.2)	20 (37.7)	60 (28.3)	22 (37.9)	69 (29.7)
Prior PCI	122 (48.2)	483 (47.7)	59 (41.5)	260 (45.8)	30 (56.6)	106 (50.5)	33 (56.9)	117 (50.4)
Prior CABG	13 (5.1)	40 (4.0)	6 (4.2)	25 (4.4)	4 (7.5)	7 (3.3)	3 (5.2)	8 (3.4)
Prior stroke	34 (13.4)	89 (8.8)	16 (11.3)	56 (9.9)	11 (20.8)	13 (6.1)	7 (12.1)	20 (8.6)
LVEF, %	50.9 ± 14.2	56.9 ± 12.3	48.4 ± 15.1	57.3 ± 12.2	52.3 ± 13.1	55.9 ± 12.9	55.9 ± 11.5	57.0 ± 12.0
Clinical presentation at the index PCI^a^								
STEMI	69 (27.5)	106 (10.5)	53 (37.9)	65 (11.5)	10 (18.9)	22 (10.4)	6 (10.3)	15 (6.5)
NSTEMI	16 (6.3)	53 (5.2)	11 (7.9)	26 (4.6)	4 (7.5)	17 (8.0)	1 (1.7)	10 (4.3)
UAP	29 (11.5)	124 (12.3)	14 (10.0)	70 (12.4)	9 (17.0)	25 (11.8)	6 (10.3)	29 (12.5)
Stable coronary artery disease	137 (54.2)	729 (72.0)	62 (44.3)	403 (71.5)	30 (56.6)	148 (69.8)	45 (77.6)	178 (76.7)
Medication at index PCI								
Aspirin	249 (98.4)	1006 (99.6)	139 (97.9)	565 (99.5)	52 (98.1)	212 (100.0)	58 (100.0)	229 (99.6)
Thienopyridine	244 (96.4)	1003 (99.1)	135 (95.1)	565 (99.5)	52 (98.1)	210 (99.1)	57 (98.3)	228 (98.3)
Anticoagulation	27 (10.7)	104 (10.3)	15 (10.6)	68 (12.0)	7 (13.2)	17 (8.0)	5 (8.6)	19 (8.3)
Beta-blocker	118 (46.6)	437 (43.3)	70 (49.3)	258 (45.4)	26 (49.1)	84 (39.6)	22 (37.9)	95 (41.3)
ACEI/ARB	142 (56.1)	621 (61.4)	80 (56.3)	357 (62.9)	30 (56.6)	132 (62.3)	32 (55.2)	132 (56.9)
Statin	173 (68.4)	769 (76.1)	94 (66.2)	447 (78.7)	38 (71.7)	158 (74.5)	41 (70.7)	164 (71.3)
Oral hypoglycemia agent	73 (28.9)	304 (30.1)	38 (26.8)	162 (28.5)	19 (35.8)	70 (33.0)	16 (27.6)	72 (31.3)
Insulin	32 (12.6)	93 (9.2)	17 (12.0)	46 (8.1)	9 (17.0)	27 (12.7)	6 (10.3)	20 (8.7)
***Lesion and procedural characteristics***								
LMCA	22 (8.7)	47 (4.6)	16 (11.3)	23 (4.0)	3 (5.7)	13 (6.1)	3 (5.2)	11 (4.7)
Proximal LAD	82 (32.4)	225 (22.2)	53 (37.3)	138 (24.3)	11 (20.8)	51 (24.1)	18 (31.0)	36 (15.5)
In-stent restenosis	37 (14.6)	97 (9.6)	13 (9.2)	45 (7.9)	10 (18.9)	25 (11.8)	14 (24.1)	27 (11.6)
Bifurcation lesion	115 (45.5)	369 (36.5)	71 (50.0)	219 (38.6)	16 (30.2)	68 (32.1)	28 (48.3)	82 (35.3)
Severe calcification	66 (26.1)	141 (13.9)	31 (21.8)	78 (13.7)	28 (52.8)	31 (14.6)	7 (12.1)	32 (13.8)
Tortuosity	59 (23.3)	189 (18.7)	25 (17.6)	94 (16.5)	13 (24.5)	39 (18.4)	21 (36.2)	56 (24.1)
Chronic total occlusion	14 (5.5)	92 (9.1)	6 (4.2)	47 (8.3)	2 (3.8)	21 (9.9)	6 (10.3)	24 (10.3)
Total stent length, mm	33.5 ± 19.5	30.5 ± 18.2	34.0 ± 18.7	29.6 ± 16.9	31.2 ± 18.0	31.3 ± 19.3	34.4 ± 22.5	32.0 ± 20.0
Stent overlap	91 (36.0)	250 (24.7)	51 (35.9)	132 (23.2)	17 (32.1)	55 (25.9)	23 (39.7)	63 (27.2)
IVUS use	185 (73.1)	668 (66.0)	102 (71.8)	376 (66.2)	44 (83.0)	140 (66.0)	39 (67.2)	152 (65.5)
OCT use	16 (6.3)	127 (12.5)	7 (4.9)	87 (15.3)	3 (5.7)	20 (9.4)	6 (10.3)	20 (8.6)
Stent edge dissection	2 (0.8)	6 (0.6)	2 (1.4)	4 (0.7)	0 (0.0)	0 (0.0)	0 (0.0)	2 (0.9)
Cancer history^a^	31 (12.3)	86 (8.5)	14 (9.9)	55 (9.7)	7 (13.2)	12 (5.7)	10 (17.2)	19 (8.2)
Currently diagnosed cancer^a^	9 (3.6)	14 (1.4)	3 (2.1)	10 (1.8)	1 (1.9)	1 (0.5)	5 (8.6)	3 (1.3)
Previously diagnosed cancer^a^	22 (8.7)	72 (7.1)	11 (7.7)	45 (7.9)	6 (11.3)	11 (5.2)	5 (8.6)	16 (6.9)
Currently treated cancer	8 (3.2)	13 (1.3)	2 (1.4)	11 (1.9)	2 (3.8)	1 (0.5)	4 (6.9)	1 (0.4)
Previously treated cancer	12 (4.7)	36 (3.6)	6 (4.2)	22 (3.9)	3 (5.7)	3 (1.4)	3 (5.2)	11 (4.7)

Values are expressed as the mean ± standard deviation or n (%)

*ACEI* angiotensin converting enzyme inhibitor, *ARB* angiotensin receptor blocker, *BMI* body mass index, *CABG* coronary artery bypass graft, *EST* early stent thrombosis, *IVUS* intravascular ultrasound, *LAD* left anterior descending, *LMCA* left main coronary artery, *LST* late stent thrombosis, *LVEF* left ventricular ejection fraction, *NSTEMI* non-ST elevation myocardial infarction, *OCT* optical coherence tomography, *PCI* percutaneous coronary intervention, *ST* stent thrombosis, *STEMI* ST elevation myocardial infarction, *UAP* unstable angina pectoris, *VLST* very late stent thrombosis

^a^Variables used for multivariable logistic analyses evaluating the predictors of each type of ST

For each group defined by the combination of the presence or absence of G2-ST and cancer, the cumulative incidence of all-cause death and other clinical endpoints from the index ST dates were estimated by the Kaplan-Meier method, and differences between groups were assessed with the log-rank test. Univariable Cox regression analysis was used to estimate the hazard ratio (HR) of each group for the study endpoints. All statistical analysis were performed using SPSS for Windows version 25 (IBM SPSS Inc., Armonk, NY, USA) and MedCalc software version 19.8 (MedCalc Software Ltd, Ostend, Belgium).

## Results

### Study population

**Fig. 1:**
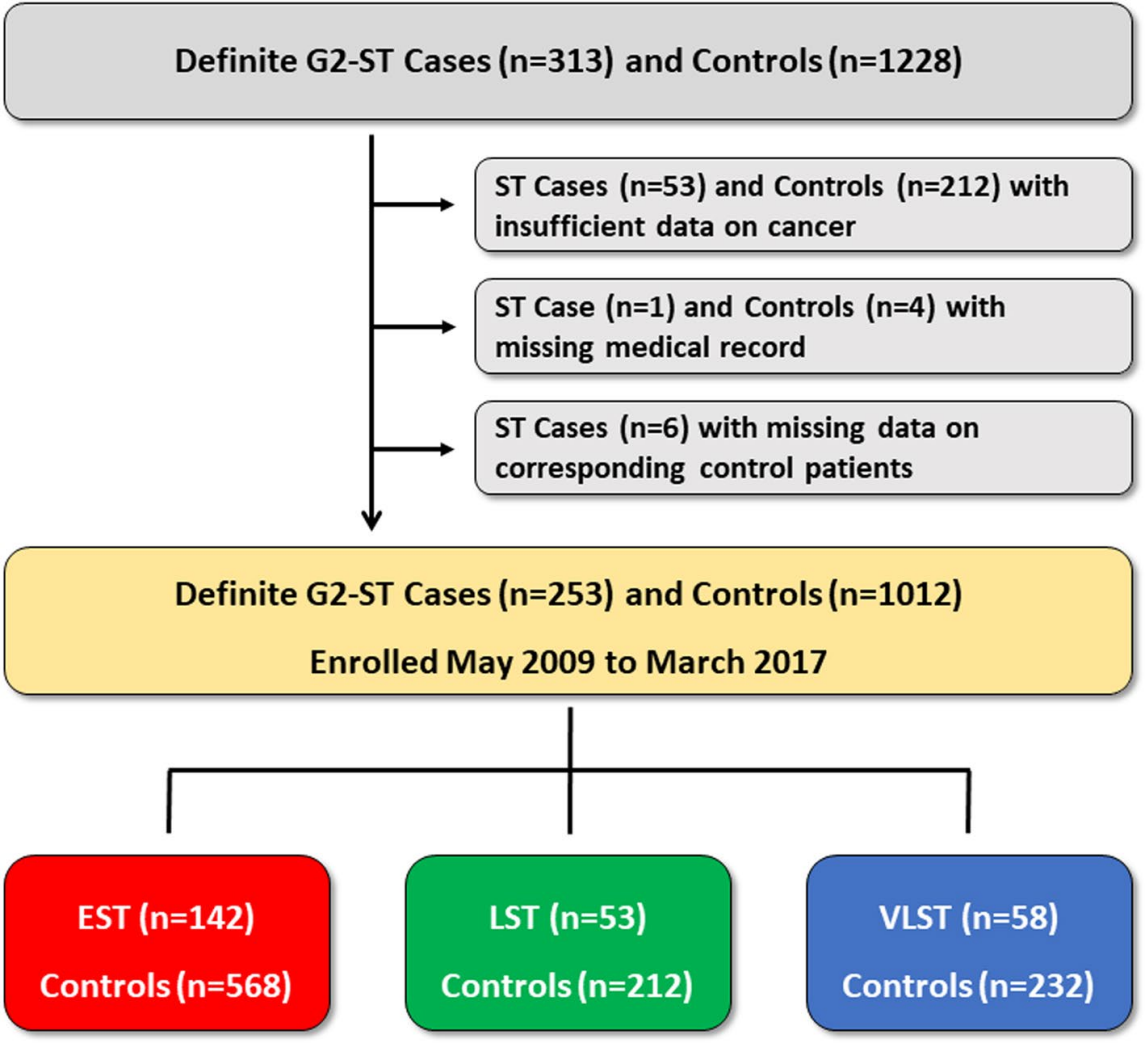
Study flowchart. EST = early stent thrombosis; G2-ST = second-generation drug-eluting stent thrombosis; LST = late stent thrombosis; ST = stent thrombosis; VLST = very late stent thrombosis

Of 1541 patients (G2-ST, *n* = 313; controls, *n* = 1228), 276 were excluded due to the following reasons: insufficient data on cancer (G2-ST, *n* = 53; controls, *n* = 212), missing medical records (G2-ST, *n* = 1; controls, *n* = 4), and missing data on corresponding control patients (G2-ST, *n* = 6). Finally, 1265 patients (G2-ST, *n* = 253; controls, *n* = 1012) were enrolled in this study (Fig. 1). Table 1 shows the baseline patient and lesion characteristics of the G2-ST cases and controls.

### Comparison of the prevalence of cancer history

**Fig. 2:**
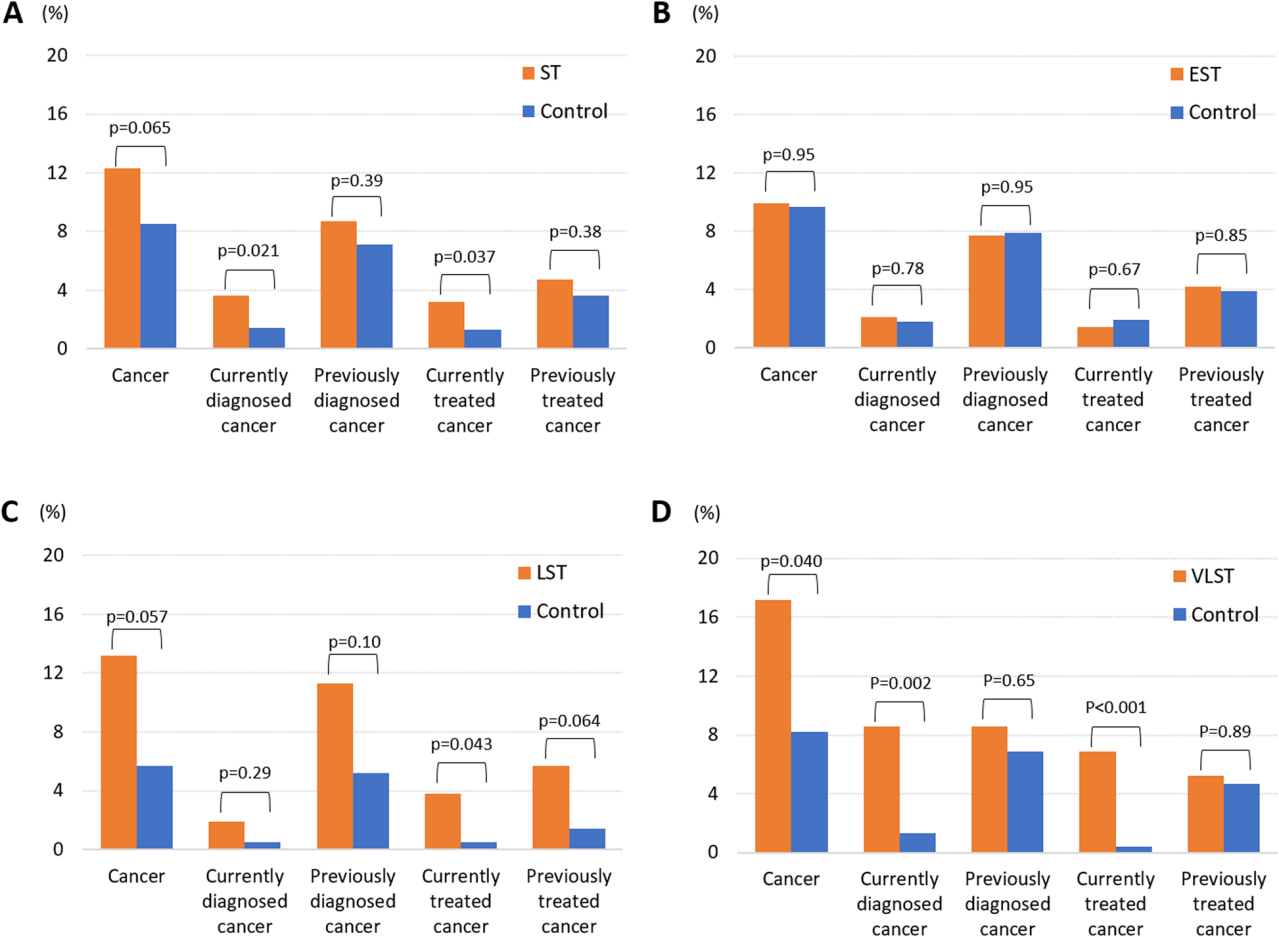
Comparisons in the prevalence of cancer history between stent thrombosis cases and controls. **A** Comparison between ST cases and their controls. **B** Comparison between EST cases and their controls. **C** Comparison between LST cases and their controls. **D** Comparison between VLST cases and their controls. EST = early stent thrombosis; LST = late stent thrombosis; ST = stent thrombosis; VLST = very late stent thrombosis

The prevalence of patients with any cancer history was higher in the G2-ST cases than in the controls (12.3% vs. 8.5%, *p* = 0.065) (Table 2 and Fig. 2A). The prevalence of patients with currently diagnosed cancer and currently treated cancer was significantly higher in G2-ST cases than in controls (3.6% vs. 1.4%, *p* = 0.021; 3.2% vs. 1.3%, *p* = 0.037, respectively) (Table 1 and Fig. 2A). According to the timing of ST, the prevalence of any cancer history was higher in LST (13.2% vs. 5.7%, *p* = 0.057) (Table 1 and Fig. 2C) and VLST cases (17.2% vs. 8.2%, *p* = 0.040) than in controls (Table 1 and Fig. 2D); whereas there was no significant difference in the prevalence of EST between G2-ST cases and controls (9.9% vs. 9.7%, *p* = 0.95)** (**Table 1 and Fig. 2B). According to the timing of cancer diagnosis or treatment, the prevalence of currently treated cancer was significantly higher in LST cases than in controls (3.8% vs. 0.5%, *p* = 0.043) (Table 1 and Fig. 2C), and the prevalence of currently diagnosed and currently treated cancer were significantly higher in VLST cases than in controls (8.6% vs. 1.3%, *p* = 0.002; 6.9% vs. 0.4%, *p* < 0.001, respectively) (Table 1 and Fig. 2D). In contrast, there was no difference between EST cases and controls in the prevalence of cancer at any timing of diagnosis or treatment (Table 1 and Fig. 2B).

**Table 2:** Detailed cancer information in ST cases and controls

	**ST (*n* = 253)**	**Controls (*n* = 1012)**
Cancer	31 (12.3)	86 (8.5)
Cancer type		
Bladder	4 (1.6)	2 (0.2)
Brain	1 (0.4)	1 (0.1)
Breast	1 (0.4)	4 (0.4)
Colon	8 (3.2)	19 (1.9)
Esophageal	2 (0.8)	1 (0.1)
Gastric	6 (2.4)	14 (1.4)
Hematopoietic	0 (0.0)	4 (0.4)
Liver	0 (0.0)	3 (0.3)
Lung	3 (1.2)	9 (0.9)
Neck	1 (0.4)	2 (0.2)
Ovarian	0 (0.0)	1 (0.1)
Pancreas	0 (0.0)	3 (0.3)
Prostate	3 (1.2)	13 (1.3)
Rectum	0 (0.0)	4 (0.4)
Renal	1 (0.4)	4 (0.4)
Skin	0 (0.0)	2 (0.2)
Thyroid	4 (1.6)	1 (0.1)
Uterine	0 (0.0)	5 (0.5)
Other	0 (0.0)	3 (0.3)
Cancer stage		
I	7 (2.8)	26 (2.6)
II	3 (1.2)	11 (1.1)
III	2 (0.8)	3 (0.3)
IV	2 (0.8)	6 (0.6)
Unknown	17 (6.7)	40 (4.0)
Metastasis	3 (1.2)	7 (0.7)
Treatment		
Surgical therapy	22 (8.7)	62 (6.1)
Radiation therapy	5 (2.0)	9 (0.9)
Chemotherapy	4 (1.6)	21 (2.1)
Cisplatin	2 (0.8)	3 (0.3)
Gemcitabine	0 (0.0)	3 (0.3)
Hormone therapy	2 (0.8)	7 (0.7)

Values are expressed as n (%). *ST*  stent thrombosis

### Detailed cancer information

Detailed cancer information (e.g., cancer type, stage, and treatment) in G2-ST cases and controls with cancer history are summarized in Table 2. Colon and gastric cancer were the major cancer types in both groups. Bladder and thyroid cancer were significantly more frequent in G2-ST cases than in controls (1.6% vs. 0.2%, *p* = 0.004; 1.6% vs. 0.1%, *p* < 0.001, respectively). Regarding cancer treatment, the frequency of patients who received surgical and radiation therapy was slightly higher in G2-ST cases than in controls (8.7% vs. 6.1%, *p* = 0.14; 2.0% vs. 0.9%, *p* = 0.14, respectively).

### Association between cancer history and G2-ST

Table 3 summarizes the logistic regression analysis results for definite ST, overall and according to ST type (EST, LST, and VLST). In model 1, any cancer history was weakly associated with overall definite ST (odds ratio [OR], 1.54; 95% confidence intervals [CI], 0.97–2.45; *p* = 0.067). In contrast, in model 2, currently diagnosed cancer (OR, 3.21; 95% CI, 1.30–7.97; *p* = 0.012) was associated with overall definite ST. Risk factors of definite ST mostly differed according to the timing of ST. Any cancer history (OR, 2.40; 95% CI, 1.02–5.65; *p* = 0.046; model 1) and currently diagnosed cancer (OR, 8.73; 95% CI, 1.92–39.7; *p* = 0.005; model 2) were associated with the occurrence of VLST. Cancer history (OR, 2.80; 95% CI, 0.92–8.55; *p* = 0.071; model 1) was also associated with the occurrence of LST. However, cancer history and currently diagnosed cancer did not significantly contribute to the occurrence of EST. The full results of the multivariable models involving the cancer-related information and other clinical factors are shown in Supplemental Table 1.

**Table 3:** Logistic regression analysis for the predictors of stent thrombosis

	**Univariable analysis**			**Multivariable model 1**			**Multivariable model 2**		
	**OR**	**95% CI**	***p*-value**	**OR**^**a**^	**95% CI**	***p*-value**	**OR**^**a**^	**95% CI**	***p*-value**
**All definite ST**									
Cancer	1.50	0.97–2.33	0.067	1.54	0.97–2.45	0.067			
Cancer type classified by the diagnosed timing (vs. non-cancer)									
Currently diagnosed cancer	2.69	1.15–6.29	0.023				3.21	1.30–7.97	0.012
Previously diagnosed cancer	1.35	0.81–2.26	0.25				1.27	0.75–2.14	0.38
**EST**									
Cancer	1.02	0.55–1.89	0.95	1.01	0.51–2.00	0.97			
Cancer type classified by the diagnosed timing (vs. non-cancer)									
Currently diagnosed cancer	1.20	0.33–4.43	0.78				0.86	0.17–4.26	0.86
Previously diagnosed cancer	0.98	0.49–1.95	0.95				1.05	0.50–2.19	0.90
**LST**									
Cancer	2.54	0.95–6.80	0.064	2.80	0.92–8.55	0.071			
Cancer type classified by the diagnosed timing (vs. non-cancer)									
Currently diagnosed cancer	4.35	0.27–70.8	0.30				9.77	0.55–172.4	0.12
Previously diagnosed cancer	2.37	0.83–6.74	0.11				2.35	0.71–7.74	0.16
**VLST**									
Cancer	2.34	1.02–5.34	0.045	2.40	1.02–5.65	0.046			
Cancer type classified by the diagnosed timing (vs. non-cancer)									
Currently diagnosed cancer	7.40	1.71–32.0	0.007				8.73	1.92–39.7	0.005
Previously diagnosed cancer	1.39	0.48–3.97	0.54				1.32	0.44–3.94	0.62

*CI* confidence interval, *EST* early stent thrombosis, *LST* late stent thrombosis, *OR* odds ratio, *ST* stent thrombosis, *VLST* very late stent thrombosis

^a^Adjusted for covariates listed in Table 1

### Clinical outcomes

**Fig. 3:**
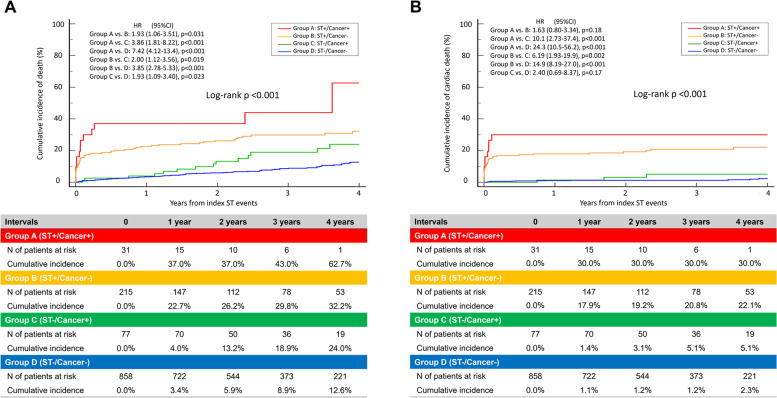
Cumulative 4-Year Incidence of All-Cause Death and Cardiac Death According to the Presence or Absence of Cancer History and Stent Thrombosis. **A** All-cause death. **B** Cardiac death. CI = confidence intervals; HR = hazard ratio; ST = stent thrombosis

**Fig. 4:**
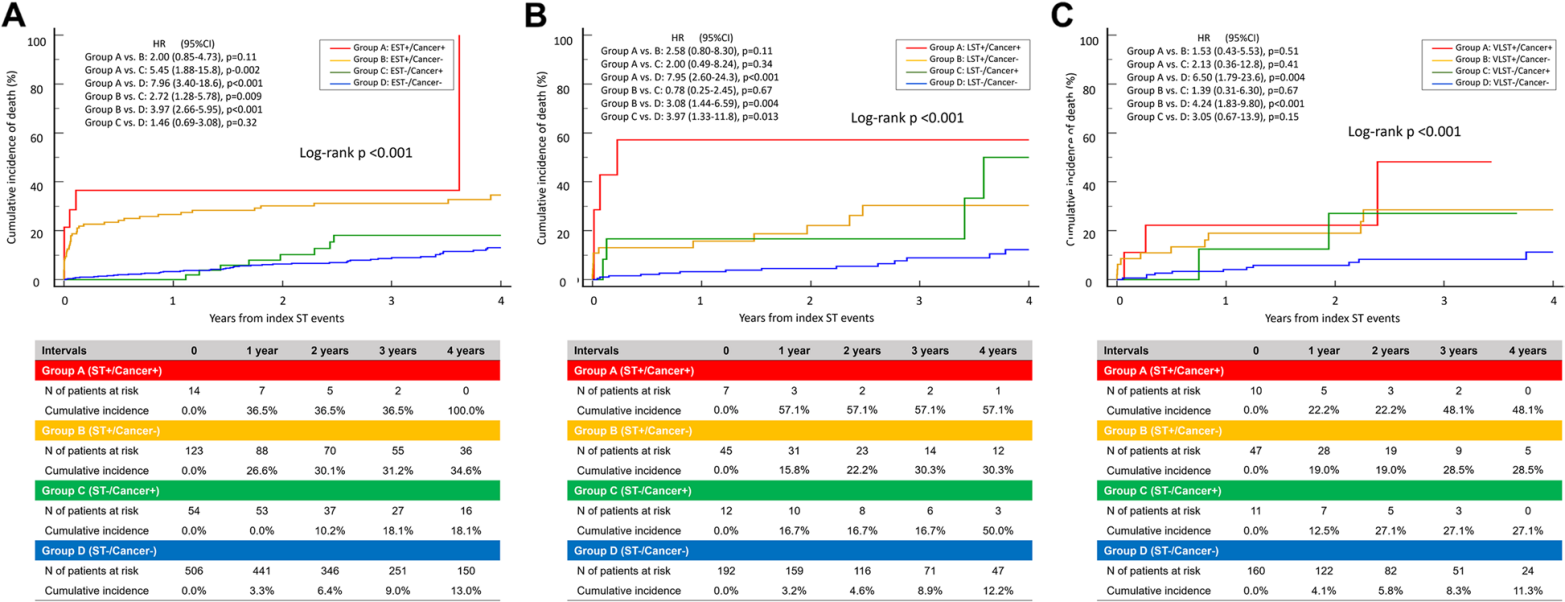
Cumulative 4-Year Incidence of All-Cause Death in Patients with and without Cancer History According to the Timing of Stent Thrombosis. **A** Comparison between EST cases and their conrols. **B** Comparison between LST cases and their controls. **C** Comparison between VLST cases and their controls. CI = confidence intervals; EST = early stent thrombosis; HR = hazard ratio; LST = late stent thrombosis; VLST = very late stent thrombosis

During the median follow-up period of 872 (452, 1386) days, all-cause death occurred in 79 patients with G2-ST and 119 control patients. The cumulative 4-year incidence of all-cause death was significantly higher in patients with cancer than in those without cancer among G2-ST cases (62.7% vs. 32.2%; HR, 1.93; 95% CI, 1.06–3.51; *p* = 0.031) and among controls (24.0% vs. 12.6%; HR, 1.93; 95% CI: 1.09–3.40; *p* = 0.023) (Fig. 3A and Table 4). The cumulative 4-year incidence of cardiac death tended to be higher in patients with cancer than in those without cancer among both G2-ST cases (30.0% vs. 22.1%; HR, 1.63; 95% CI, 0.80–3.34; *p* = 0.18) and controls (5.1% vs. 2.3%; HR, 2.40; 95% CI, 0.69–8.37; *p* = 0.17) **(**Fig. 3B and Table 4). Figure 4 shows the mortality according to ST type and cancer history. Cumulative incidences of individual outcomes are summarized in Table 4. Among G2-ST cases, the cumulative 4-year incidence of non-cardiac death and MACE was significantly higher in patients with cancer than in those without cancer (46.7% vs. 8.9%; HR, 3.05; 95% CI, 1.01–9.21; *p* = 0.048; 67.9% vs. 36.0%; HR, 1.96; 95% CI, 1.12–3.41; *p* = 0.018, respectively). Similarly, among controls, non-cardiac death and MACE occurred more frequently in patients with cancer than in those without cancer (19.8% vs. 8.7%; HR, 2.24; 95% CI, 1.17–4.29; *p* = 0.015; 28.4% vs. 14.9%; HR, 1.97; 95% CI, 1.19–3.25; *p* = 0.008, respectively). Other outcomes did not differ between groups.

**Table 4:** Clinical Events throughout the Entire Follow-up Period

	**ST (*n* = 253)**				**Controls (*n* = 1012)**			
	**Patients with events (Cumulative 4-year incidence)**		**HR (95% CI)**	***p*-value**	**Patients with events (Cumulative 4-year incidence)**		**HR (95% CI)**	***p*-value**
	**Cancer** **(*n* = 31)**	**Non-cancer** **(*n* = 222)**			**Cancer** **(*n* = 86)**	**Non-cancer** **(*n* = 926)**		
All-cause death	13 (62.7%)	66 (32.2%)	1.93 (1.06–3.51)	0.031	19 (24.0%)	100 (12.6%)	1.93 (1.09–3.40)	0.023
Cardiac death	9 (30.0%)	47 (22.1%)	1.63 (0.80–3.34)	0.18	4 (5.1%)	18 (2.3%)	2.40 (0.69–8.37)	0.17
Non-cardiac death	4 (46.7%)	15 (8.9%)	3.05 (1.01–9.21)	0.048	14 (19.8%)	66 (8.7%)	2.24 (1.17–4.29)	0.015
Non-fatal MI	3 (12.5%)	15 (8.6%)	1.89 (0.54–6.55)	0.32	1 (1.4%)	10 (1.2%)	1.13 (0.14–8.84)	0.91
TLR	4 (34.3%)	42 (27.4%)	0.99 (0.36–2.78)	0.99	8 (13.1%)	74 (7.8%)	1.13 (0.49–2.61)	0.78
MACE	15 (67.9%)	77 (36.0%)	1.96 (1.12–3.41)	0.018	23 (28.4%)	123 (14.9%)	1.97 (1.19–3.25)	0.008
Recurrent ST	2 (7.6%)	11 (5.5%)	1.41 (0.31–6.36)	0.66	-	-	-	NA
Bleeding	1 (12.5%)	4 (2.6%)	2.58 (0.29–23.3)	0.40	6 (6.1%)	24 (2.5%)	2.13 (0.79–5.78)	0.14

Values are n (%), unless otherwise indicated. The number of patients with an event was counted until the end of follow up. Cumulative 4-year incidence was estimated by the Kaplan–Meier method. HRs with 95% CIs of the cancer group relative to the non-cancer group for the outcome measures were estimated throughout the entire follow-up period by the Cox proportional hazards models

*CI* confidence interval, *HR* hazard ratio, *MACE* major adverse cardiovascular events, *MI* myocardial infarction, *ST* stent thrombosis, *TLR* target lesion revascularization

## Discussion

The main findings of the current study can be summarized as follows: (1) the prevalence of patients with any cancer history was numerically higher in G2-ST cases than in controls; (2) currently diagnosed and currently treated cancer were more frequently observed in G2-ST cases than in controls; (3) cancer history was associated with LST and VLST, but not with EST; and (4) patients with cancer history showed a higher mortality than those without cancer, regardless of the presence of G2-ST.

Since first published by Bouillaud et al. in 1823, and reported by Professor Armand Trousseau in 1865, numerous studies have reported an association between cancer and thromboembolic events [3, 4, 12]. Several advances in cancer screening, diagnosis, and treatment currently allow patients to survive longer. However, along with improved survival in cancer patients, cancer-associated thrombosis appears to be increasing over the last decade; it now emerges as the second-leading cause of death in these patients and profoundly impacts their quality of life [4, 13, 14]. Among cancer-associated thrombosis cases, venous thromboembolism represents a major cause of morbidity and mortality. Recently, Kwok et al. revealed that cancer patients had a significantly increased risk for 90-day readmission due to acute MI than non-cancer patients [15]. Furthermore, Tabata et al. reported that cancer patients had a significantly higher probability of TLR within one year after PCI than non-cancer patients [16]. These studies suggest that arterial thromboembolism is also prevalent in cancer patients, but has not been characterized as well as venous thromboembolism.

ST is rare but remains an unsolved issue in the DES era because of the high incidences of death and MI [8, 17]. The ADAPT-DES (Assessment of Dual Antiplatelet Therapy With Drug-Eluting Stents) registry, with approximately 70% of patients undergoing G2-DES implantation, demonstrated that the 2-year incidence of definite ST in patients undergoing index-PCI for MI was 1.3%, which was similar to the results of bare metal stents (BMS) or G1-DES [18]. Regarding the association between cancer and BMS-ST, the Dutch Stent Thrombosis Registry, including two-thirds of patients undergoing BMS implantation, demonstrated that malignancy was associated with the occurrence of ST [19]. However, to date, little evidence is available regarding the relationship between cancer and G2-ST, primarily due to difficulties in collecting large numbers of G2-ST cases with comprehensive data on clinical, procedural, and diagnostic variables, as well as detailed cancer information for the entire samples. To our knowledge, the current study is the first to investigate the association between cancer and G2-ST by analyzing data from a large-scale ST registry. Compared to that in controls, the prevalence of cancer history was higher in G2-ST patients, especially those with currently diagnosed and currently treated cancer. These findings indicate the potential association of cancer with the occurrence of G2-ST.

The pathophysiology of ST is multifactorial and includes patient-, lesion-, procedural-, and stent-related factors [17]. Although the underlying mechanism of ST occurrence in cancer patients remains unclear, several possible mechanisms linking cancer and ST exist. Cancer itself expresses or releases several factors directly activating the coagulation system and platelet activity [12]. Additionally, cancer cells synthesize and secrete various inflammatory cytokines, which promote endothelial damage and increase microvasculature permeability to pro-coagulating factors, inducing further progression of endothelial dysfunction and procoagulant release [20, 21]. In the present study, patients with currently diagnosed and currently treated cancer were more frequently observed in G2-ST cases than in controls, although the prevalence of patients with previously diagnosed and previously treated cancer was similar between groups. Furthermore, currently diagnosed cancer was associated with the occurrence of G2-ST. Given these findings, cancer-associated G2-ST might be attributable to an activated coagulant system, increased platelet activity, and endothelial dysfunction by active cancer. Another potential mechanism for cancer-associated G2-ST may be related to the cancer treatment. Radiation therapy and chemotherapy (e.g., cisplatin, immunomodulatory drugs, and antiangiogenic drugs) have increased the risk for venous and arterial thromboembolism [22–24]. In the present study, radiation therapy and cisplatin therapy were more frequently used in G2-ST patients than in controls, although the difference did not reach statistical significance. Considering these findings, cancer- and treatment-related factors might contribute to G2-ST occurrence in cancer patients. Further studies are required to validate our hypothesis.

Risk factors associated with ST mostly differ according to the timing of ST, regardless of the use of G1- or G2-DES [8, 17]. However, whether the relationship between cancer history and ST varies according to the timing of ST remains poorly understood. In the present study, the prevalence of currently treated cancer was significantly higher in LST cases than in controls; additionally, patients with currently diagnosed and currently treated cancer were more frequently observed in VLST cases than in controls. Intriguingly, cancer history and currently diagnosed cancer were associated with LST and VLST occurrence, but not with EST. Delayed arterial healing, characterized by poor endothelial coverage and local fibrin deposition, and neo-atherosclerosis are considered the primary substrates for LST and VLST [25, 26]. Notably, these factors are accelerated by the local inflammatory reaction after stent implantation. As systemic inflammation induced by cancer partially causes cancer-associated thrombosis, these findings may help explain the difference in the relationship between cancer history and G2-ST according to the timing of ST. Recently, Guo et al. demonstrated that the most critical period for ST in cancer patients was in the first year after PCI (i.e., EST, 20%; LST, 52%) [27]. Since the present study identified cancer history at the time of ST occurrence, the differences in the incidence rate and most frequent timing of ST between cancer and non-cancer patients could not be assessed. As such, it should be noted that the present study results did not mean that LST and VLST occurred more frequently than EST in cancer patients.

Appropriate management of cancer-associated thrombosis is crucial in improving outcomes for cancer patients. Current guidelines recommend that antithrombotic therapy (e.g., direct oral anticoagulant and low molecular weight heparin) for the prevention and treatment of venous thromboembolism should be selected based on several aspects, including the type of cancer, individual bleeding and thrombotic risk, and drug-drug interactions [28, 29]. However, limited evidence exists regarding the management of arterial thromboembolism. Even in patients with cancer, coronary revascularization is imperative in critical settings (e.g., acute coronary syndrome and uncontrollable angina despite medical therapy). However, physicians often confront a dilemma in determining the antithrombotic therapy regimen after the procedure, as these patients are likely to have both bleeding and thrombotic risk [28, 30]. Potts et al. reported that, in 6,571,034 patients undergoing PCI, 1.8% had currently diagnosed cancer and 5.8% had previous cancer; current cancer was associated with a higher rate of in-hospital bleeding than previous cancer and no cancer history [31]. Similarly, the present study found that currently diagnosed and currently treated cancer carried an increased risk of LST and VLST. Although the optimal management of arterial thromboembolism in cancer patients could not be identified in the present study, more careful follow-up is warranted in those with active cancer undergoing DES implantation.

### Limitations

The present study has several limitations. First, this study was a post hoc analysis of the REAL-ST registry; a selection bias was therefore unavoidable. Second, we retrospectively sought to collect cancer-related information from all G2-ST patients and corresponding controls. However, a relatively large number of patients (265 patients) were excluded due to insufficient information on cancer. Additionally, we might have overlooked some cases of cancer history since additional data was mainly collected from a review of medical records; this might have biased the conclusions. Third, a lack of information on medical treatment (e.g., antithrombotic therapy) at the index ST date and the case–control study design made it difficult to identify the optimal antithrombotic therapy after G2-DES implantation for cancer patients. Fourth, the case–control sampling scheme of the REAL-ST registry forced us to compare clinical outcomes (e.g., mortality) after the index ST date between cancer and non-cancer groups in G2-ST cases and controls separately. Because we showed a possible effect of cancer on G2-ST incidence in our primary analysis, stratifying G2-ST/controls may have (1) partially blocked the overall impact of cancer on clinical outcomes through G2-ST and (2) induced collider (i.e., common effect)-stratification bias through unmeasured risk factors for G2-ST and clinical outcomes [32]. However, as these biases would likely underestimate the risk increase due to cancer, our results still suggest that cancer is a significant risk factor for managing patients’ prognosis following G2-DES implantation. Fifth, the risk of death might not be negligible among cancer patients, whereas the competing-risk survival analysis methods (e.g., multi-state models and the Fine-Gray models) are not applicable to our data due to the case–control sampling design for ST onset. However, our sampling design did not exclude controls who had died before an index ST date of their case. Hence, our odds ratios estimates would approximate the sub-distribution hazard ratios that treat deaths as competing events, where those who had died remained at risk after their deaths. Finally, extrapolating our results to populations outside of Japan requires caution because the study population consisted solely of Japanese individuals.

## Conclusions

This post hoc analysis of the REAL-ST registry revealed that G2-ST patients had a higher prevalence of currently diagnosed and currently treated cancer than controls. Notably, cancer history was associated with the occurrence of LST and VLST, but not with EST.

## Supplementary Information

**Additional file 1: Supplementary Appendix. **List of Participating Centers and Investigators. **Supplemental Table 1. **Logistic Regression Analysis for the Predictors of ST. 

## Data Availability

The data, analytic methods, and study materials will not be made available to other researchers for purposes of reproducing the results or replicating the procedure because of the legal regulations about access to patient-level data.

## Abbreviations

BMS: Bare metal stents
EST: Early stent thrombosis
G1-DES: First-generation drug-eluting stents
G2-DES: Second-generation drug-eluting stents
G2-ST: Second-generation drug-eluting stent thrombosis
LST: Late stent thrombosis
REAL-ST: Retrospective multicenter registry of patients with ST after G1-and G2-DES implantation
ST: Stent thrombosis
VLST: Very late stent thrombosis
