# Supplementary material for: Association between cancer history and second-generation drug-eluting stent thrombosis: insights from the REAL-ST registry
Source: Thromb J. 2023 May 24;21:60. doi: 10.1186/s12959-023-00503-5 (PMC10207799; doi:10.1186/s12959-023-00503-5)
Supplement: Supplementary file 1 — Additional file 1: Supplementary Appendix. List of Participating Centers and Investigators. Supplemental Table 1. Logistic Regression Analysis for the Predictors of ST. [file 12959_2023_503_MOESM1_ESM.docx]

**SUPPLEMENTAL MATERIAL**

**Article title: Association Between Cancer History and Second-Generation Drug-Eluting Stent Thrombosis: Insights from the REAL-ST Registry**

**Authors: Hamana et al.**

**TABLE OF CONTENTS**

**Appendix**:

List of Participating Centers and Investigators …. p2

**Supplemental Table 1**:

Logistic Regression Analysis for the Predictors of ST p5

**Supplementary Appendix. List of Participating Centers and Investigators**

Chidoribashi Hospital: Fumitoshi Toyota, Yohei Sasaki

Fujimoto General Hospital: Hideaki Otsuji

Fukuoka University: Makoto Sugihara, Makito Futami

Fukuoka Wajiro Hospital: Takeshi Serikawa

Gifu Heart Center: Hitoshi Matsuo, Toru Tanigaki

Gifu Prefectural General Medical Center: Toshiyuki Noda, Takashi Kato

Hiroshima City Hiroshima Citizens Hospital: Kazuoki Dai

Hyogo Prefectural Awaji Medical Center: Masamichi Iwasaki

Hyogo Prefectural Himeji Cardiovascular Center: Tomofumi Takaya

Ichinomiyanishi Hospital: Kazuhiro Dan, Kei Ichihashi

Izumi Regional Medical Center: Hideto Okino

Japanese Red Cross Wakayama Medical Center: Mamoru Toyofuku

Kawakita General Hospital: Atsushi Tosaka

Kindai University Faculty of Medicine: Gaku Nakazawa

Kitaishikai Hospital: Makoto Saito

Kobe City Medical Center General Hospital: Kite Kim

Kobe University: Hiromasa Otake, Akira Nagasawa, Tomoyo Hamana

Kokura Memorial Hospital: Kenji Ando, Shoichi Kuramitsu

Kurashiki Central Hospital: Kazushige Kadota, Masanobu Ohya

Kurume University: Takaharu Nakayoshi, Hidetoshi Chibana

Kyoto University: Takeshi Kimura, Hiroki Shiomi

Megumino Hospital: Yoshinori Shimooka

Miyazaki Medical Association Hospital Cardiovascular Center: Yoshisato Shibata, Kenji Ogata

Miyazaki Prefectural Nobeoka Hospital: Kazumasa Kurogi

Nakadori General Hospital: Ryohei Sakamoto

National Hospital Organization Kagoshima Medical Center: Tetsuro Kataoka

National Hospital Organization Kyoto Medical Center: Mitsuru Ishii

National Hospital Organization Ureshino Medical Center: Fumi Yamamoto

New Tokyo Hospital: Hiroyoshi Kawamoto, Hiroto Yabushita

Osaka Saiseikai Nakatsu Hospital: Amane Kozuki

Osaka Red Cross Hospital: Yohei Kobayashi

Otsu Red Cross Hospital: Hirooki Higami

Saiseikai Kumamoto Hospital: Hiroto Suzuyama

Saitama Medical Center, Jichi Medical University: Kenichi Sakakura, Yusuke Watanabe

Saga University: Shinjo Sonoda, Masahiro Natsuaki

Sapporo Higashi Tokushukai Hospital: Seiji Yamasaki, Yuki Katagiri

Sendai Kosei Hospital: Norio Tada, Kazunori Horie

Sendai Open Hospital: Toru Takii

Shonan Kamakura General Hospital: Shigeru Saito, Futoshi Yamanaka

Takagi Hospital: Daigo Mine

Tenri Hospital: Soichiro Enomoto

Tokai University: Shingo Matsumoto

Tokeidai Memorial Hospital: Takuya Haraguchi

Tokyo Medical University Hachioji Medical Center: Nobuhiro Tanaka

Tsukuba Medical Center Hospital: Hidetaka Nishina, Yuki Kakefuda

University of Occupational and Environmental Health Japan School of Medicine: Reo Anai

Yamato Seiwa Hospital: Tatsuki Doijiri

**Supplemental Table 1. Logistic Regression Analysis for the Predictors of ST**

|  | **Univariate analysis** | | | **Multivariate model 1** | | | **Multivariate model 2** | | |
| --- | --- | --- | --- | --- | --- | --- | --- | --- | --- |
|  | **OR** | **95% CI** | **p-value** | **OR*** | **95% CI** | **p-value** | **OR*** | **95% CI** | **p-value** |
| **All definite ST** | | | | | | | | | |
| Age | 0.98 | 0.97-1.00 | 0.018 | 0.99 | 0.98-1.01 | 0.31 | 0.99 | 0.98-1.01 | 0.31 |
| Male sex | 1.09 | 0.79-1.52 | 0.60 | 0.97 | 0.68-1.37 | 0.85 | 0.95 | 0.67-1.35 | 0.77 |
| Clinical presentation at the index PCI (vs. stable angina) | | | | | | | | | |
| STEMI | 3.60 | 2.52-5.14 | <0.001 | 3.67 | 2.54-5.31 | <0.001 | 3.75 | 2.59-5.43 | <0.001 |
| NSTEMI | 1.61 | 0.89-2.89 | 0.11 | 1.76 | 0.97-3.19 | 0.065 | 1.77 | 0.98-3.22 | 0.061 |
| UAP | 1.24 | 0.80-1.94 | 0.33 | 1.21 | 0.77-1.90 | 0.41 | 1.23 | 0.78-1.94 | 0.36 |
| Hemodialysis | 2.27 | 1.50-3.46 | <0.001 | 2.49 | 1.59-3.92 | <0.001 | 2.58 | 1.64-4.06 | <0.001 |
| Hypertension | 0.83 | 0.58-1.17 | 0.27 | 0.87 | 0.61-1.26 | 0.47 | 0.85 | 0.59-1.23 | 0.40 |
| Dyslipidemia | 0.93 | 0.65-1.34 | 0.71 | 0.89 | 0.60-1.33 | 0.56 | 0.89 | 0.60-1.34 | 0.59 |
| Current smoking | 1.45 | 1.06-1.99 | 0.022 | 1.33 | 0.94-1.89 | 0.11 | 1.30 | 0.92-1.85 | 0.14 |
| Diabetes mellitus | 1.19 | 0.90-1.56 | 0.23 | 1.14 | 0.85-1.52 | 0.38 | 1.14 | 0.85-1.53 | 0.37 |
| Cancer | 1.50 | 0.97-2.33 | 0.067 | 1.54 | 0.97-2.45 | 0.067 | - | - | - |
| Cancer type classified by the diagnosed timing (vs. non-cancer) | | | | | | | | | |
| Currently diagnosed cancer | 2.69 | 1.15-6.29 | 0.023 | - | - | - | 3.21 | 1.30-7.97 | 0.012 |
| Previously diagnosed cancer | 1.35 | 0.81-2.26 | 0.25 | - | - | - | 1.27 | 0.75-2.14 | 0.38 |
| **EST** | | | | | | | | | |
| Age | 0.99 | 0.97-1.00 | 0.11 | 1.00 | 0.98-1.02 | 0.96 | 1.00 | 0.98-1.02 | 0.96 |
| Male sex | 1.41 | 0.89-2.23 | 0.15 | 1.25 | 0.76-2.06 | 0.39 | 1.25 | 0.76-2.06 | 0.38 |
| Clinical presentation at the index PCI (vs. stable angina) | | | | | | | | | |
| STEMI | 5.30 | 3.38-8.32 | <0.001 | 5.49 | 3.43-8.79 | <0.001 | 5.48 | 3.42-8.77 | <0.001 |
| NSTEMI | 2.75 | 1.29-5.85 | 0.009 | 2.99 | 1.39-6.44 | 0.005 | 2.99 | 1.39-6.45 | 0.005 |
| UAP | 1.30 | 0.69-2.45 | 0.42 | 1.30 | 0.68-2.47 | 0.42 | 1.30 | 0.68-2.46 | 0.43 |
| Hemodialysis | 1.13 | 0.53-2.43 | 0.75 | 1.49 | 0.66-3.40 | 0.34 | 1.48 | 0.65-3.39 | 0.35 |
| Hypertension | 0.79 | 0.50-1.25 | 0.31 | 0.84 | 0.51-1.38 | 0.49 | 0.84 | 0.51-1.39 | 0.50 |
| Dyslipidemia | 0.94 | 0.57-1.55 | 0.80 | 0.71 | 0.41-1.24 | 0.23 | 0.71 | 0.41-1.24 | 0.23 |
| Current smoking | 1.59 | 1.06-2.38 | 0.025 | 1.41 | 0.90-2.21 | 0.13 | 1.42 | 0.90-.2.22 | 0.13 |
| Diabetes mellitus | 1.15 | 0.80-1.67 | 0.45 | 1.24 | 0.83-1.84 | 0.29 | 1.23 | 0.83-1.84 | 0.30 |
| Cancer | 1.02 | 0.55-1.89 | 0.95 | 1.01 | 0.51-2.00 | 0.97 | - | - | - |
| Cancer type classified by the diagnosed timing (vs. non-cancer) | | | | | | | | | |
| Currently diagnosed cancer | 1.20 | 0.33-4.43 | 0.78 | - | - | - | 0.86 | 0.17-4.26 | 0.86 |
| Previously diagnosed cancer | 0.98 | 0.49-1.95 | 0.95 | - | - | - | 1.05 | 0.50-2.19 | 0.90 |
| **LST** | | | | | | | | | |
| Age | 0.98 | 0.95-1.00 | 0.091 | 0.98 | 0.95-1.01 | 0.23 | 0.98 | 0.95-1.01 | 0.23 |
| Male sex | 0.88 | 0.43-1.77 | 0.71 | 0.78 | 0.34-1.76 | 0.54 | 0.75 | 0.33-1.71 | 0.50 |
| Clinical presentation at the index PCI (vs. stable angina) | | | | | | | | | |
| STEMI | 2.24 | 0.96-5.22 | 0.061 | 1.77 | 0.68-4.60 | 0.24 | 1.80 | 0.69-4.70 | 0.23 |
| NSTEMI | 1.16 | 0.36-3.69 | 0.80 | 1.38 | 0.39-4.86 | 0.61 | 1.45 | 0.41-5.13 | 0.56 |
| UAP | 1.78 | 0.75-4.18 | 0.19 | 1.75 | 0.67-4.54 | 0.25 | 1.80 | 0.69-4.68 | 0.23 |
| Hemodialysis | 5.67 | 2.80-11.5 | <0.001 | 6.37 | 2.91-13.9 | <0.001 | 6.53 | 2.97-14.3 | <0.001 |
| Hypertension | 0.70 | 0.33-1.46 | 0.34 | 0.54 | 0.23-1.27 | 0.16 | 0.53 | 0.23-1.24 | 0.14 |
| Dyslipidemia | 0.84 | 0.37-1.89 | 0.67 | 1.06 | 0.41-2.76 | 0.91 | 1.04 | 0.40-2.72 | 0.93 |
| Current smoking | 1.29 | 0.57-2.92 | 0.54 | 1.39 | 0.54-3.60 | 0.49 | 1.41 | 0.55-3.64 | 0.48 |
| Diabetes mellitus | 1.62 | 0.87-3.00 | 0.13 | 1.39 | 0.71-2.75 | 0.34 | 1.36 | 0.69-2.69 | 0.37 |
| Cancer | 2.54 | 0.95-6.80 | 0.064 | 2.80 | 0.92-8.55 | 0.071 | - | - | - |
| Cancer type classified by the diagnosed timing (vs. non-cancer) | | | | | | | | | |
| Currently diagnosed cancer | 4.35 | 0.27-70.8 | 0.30 | - | - | - | 9.77 | 0.55-172.4 | 0.12 |
| Previously diagnosed cancer | 2.37 | 0.83-6.74 | 0.11 | - | - | - | 2.35 | 0.71-7.74 | 0.16 |
| **VLST** | | | | | | | | | |
| Age | 0.99 | 0.96-1.02 | 0.42 | 0.98 | 0.95-1.02 | 0.30 | 0.98 | 0.95-1.02 | 0.29 |
| Male sex | 0.77 | 0.40-1.46 | 0.42 | 0.66 | 0.33-1.30 | 0.23 | 0.62 | 0.31-1.23 | 0.17 |
| Clinical presentation at the index PCI (vs. stable angina) | | | | | | | | | |
| STEMI | 1.58 | 0.58-4.31 | 0.37 | 1.73 | 0.62-4.86 | 0.30 | 1.92 | 0.68-5.41 | 0.21 |
| NSTEMI | 0.40 | 0.05-3.17 | 0.38 | 0.41 | 0.05-3.41 | 0.41 | 0.41 | 0.05-3.46 | 0.41 |
| UAP | 0.82 | 0.32-2.09 | 0.68 | 0.91 | 0.35-2.38 | 0.85 | 0.98 | 0.37-2.57 | 0.97 |
| Hemodialysis | 1.79 | 0.74-4.33 | 0.19 | 1.91 | 0.73-5.04 | 0.19 | 2.09 | 0.79-5.51 | 0.14 |
| Hypertension | 1.09 | 0.51-2.33 | 0.82 | 1.15 | 0.52-2.54 | 0.72 | 1.10 | 0.50-2.44 | 0.82 |
| Dyslipidemia | 1.00 | 0.49-2.03 | 1.00 | 1.08 | 0.49-2.38 | 0.85 | 1.00 | 0.45-2.23 | 0.99 |
| Current smoking | 1.25 | 0.63-2.48 | 0.52 | 1.21 | 0.55-2.66 | 0.64 | 1.06 | 0.47-2.41 | 0.88 |
| Diabetes mellitus | 0.97 | 0.54-1.72 | 0.91 | 0.87 | 0.47-1.61 | 0.67 | 0.85 | 0.46-1.57 | 0.59 |
| Cancer | 2.34 | 1.02-5.34 | 0.045 | 2.40 | 1.02-5.65 | 0.046 | - | - | - |
| Cancer type classified by the diagnosed timing (vs. non-cancer) | | | | | | | | | |
| Currently diagnosed cancer | 7.40 | 1.71-32.0 | 0.007 | - | - | - | 8.73 | 1.92-39.7 | 0.005 |
| Previously diagnosed cancer | 1.39 | 0.48-3.97 | 0.54 | - | - | - | 1.32 | 0.44-3.94 | 0.62 |

CI = confidence interval; EST = early stent thrombosis; LST = late stent thrombosis; NSTEMI = non-ST elevation myocardial infarction; OR = odds ratio; ST = stent thrombosis; STEMI = ST elevation myocardial infarction; UAP = unstable angina pectoris; VLST = very late stent thrombosis.

*Adjusted for covariates listed in **Table 1**.
